# Chylothorax Chronicles: Tubercular Lymphadenopathy and Hemophagocytic Lymphohistiocytosis

**DOI:** 10.7759/cureus.53271

**Published:** 2024-01-31

**Authors:** Souvik Sarkar, Ulhas Jadhav, Pankaj Wagh, Babaji Ghewade

**Affiliations:** 1 Respiratory Medicine, Jawaharlal Nehru Medical College, Datta Meghe Institute of Medical Sciences, Deemed to be University, Wardha, Wardha, IND

**Keywords:** multi-disciplinary team, thoracic duct, thoracic duct embolization, rare cause of pleural effusion, hemophagocytic lymphohistiocytosis (hlh), tuberculous adenopathy, bilateral chylothorax

## Abstract

Chylothorax delineates a state marked by the accumulation of chyle, an opalescent fluid laden with lipids, within the pleural cavity. This occurrence commonly ensues from the seepage of chyle originating from the thoracic duct, occasioned by trauma, surgical interventions, or underlying pathological conditions. This phenomenon induces respiratory distress, necessitating intricate and tailored interventions for its resolution. In this report, we present the case of a 27-year-old male who was admitted with a two-month history of symptoms, including dry cough, weakness, weight loss, and intermittent fever. Previously treated for pleural effusions and ascites, he was referred to our hospital with an intercostal drainage tube in place. Initial examinations revealed respiratory distress, fever, and bilateral pleural effusions. Laboratory results and fluid analysis indicated significant abnormalities, prompting further investigations, including CT scans and biopsies. The patient was diagnosed with chylothorax with chylous ascites due to abdominal tubular lymphadenopathy and hemophagocytic lymphohistiocytosis (HLH) and started on anti-tubercular therapy (AKT4) and octreotide. The patient was also initially managed with non-invasive ventilatory (NIV) support, intravenous antibiotics, nebulizations, an intercostal chest drain (ICD), and a thoracic duct embolization (TDE). Regular monitoring and collaboration between specialties were crucial, ultimately resulting in the removal of the drainage tube and the patient's stable discharge.

## Introduction

A lipid-based effusion may present as either chylothorax or cholesterol pleural effusion, frequently displaying a milky appearance attributed to elevated lipid concentration. Despite this similarity, they significantly differ in pathogenesis, clinical presentation, diagnosis, predisposing conditions, and management [1]. Diagnosing chylothorax involves analyzing pleural fluid, with elevated triglyceride levels (>110 mg/dL) and the presence of chylomicrons being indicative. Management often begins with conservative measures, including dietary modifications and chest tube drainage. If conservative approaches fail, procedures like thoracic duct embolization may be considered. Intractable cases may be addressed with surgical measures, including procedures like thoracic duct ligation. Collaboration between specialties, including respiratory, surgical, and nutritional support, is crucial for comprehensive care and the successful management of chylothorax. Regular monitoring and a tailored approach are essential for optimal patient outcomes.

## Case presentation

A 27-year-old man sought admission to our medical facility, manifesting two-month-long complaints of a dry cough, pervasive lethargy, substantial weight decline, and sporadic bouts of fever. Having undergone a 15-day treatment at an alternate institution for pleural effusion and ascites, he was subsequently referred to our hospital post introduction of an intercostal drainage (ICD) tube to relieve the effusion. Following a thorough examination, the patient showed full consciousness and orientation, along with a respiratory rhythm of 40 cycles per minute. Clinical indicators included pyrexia at 38.5℃, tachycardia registering at 120 beats per minute, and a normotensive state with blood pressure measured at 110/80 millimeters of mercury. Auscultation of the chest revealed bilaterally diminished breath sounds. Abdominal examination revealed shifting dullness and edema over the bilateral lower extremities.

**Figure 1:**
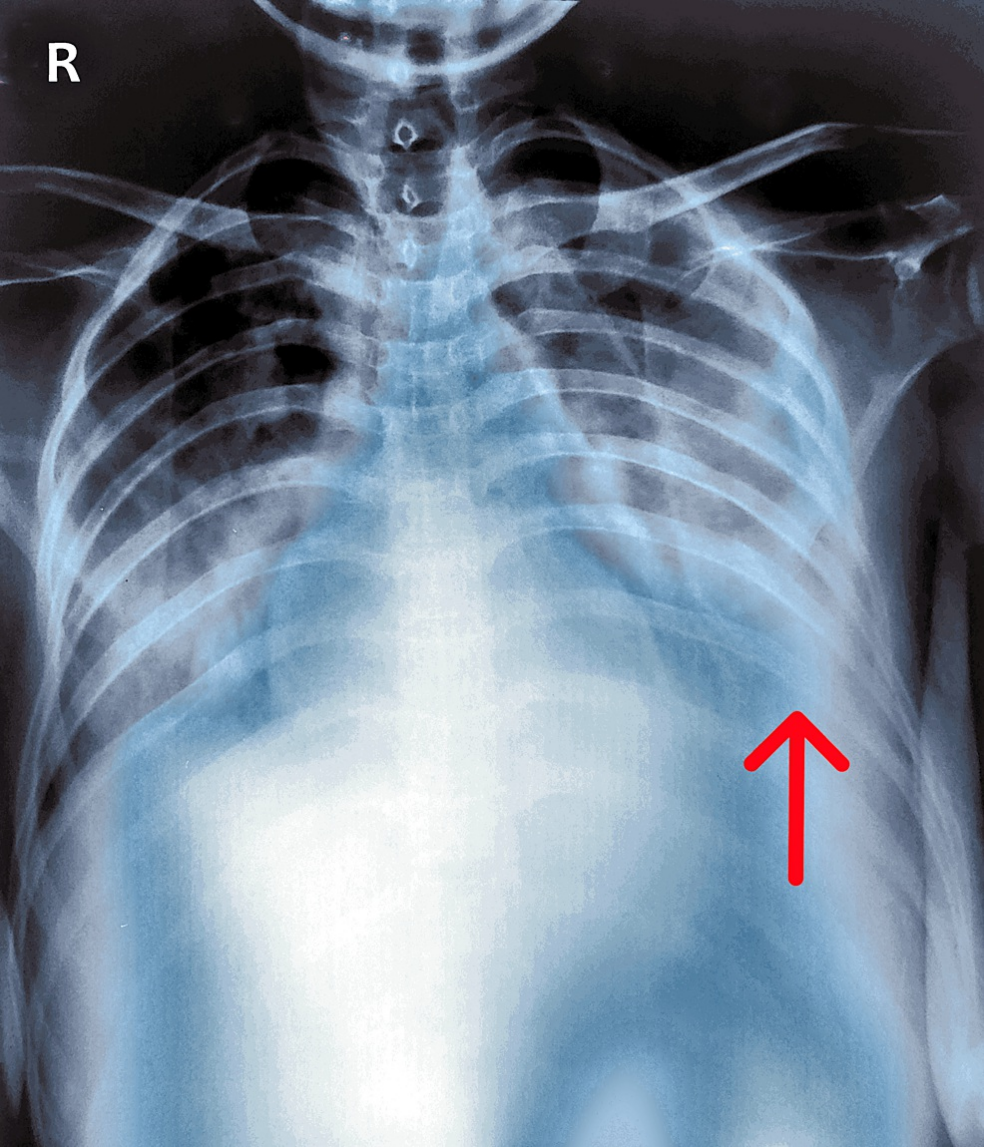
A chest x-ray anterior-posterior view showing heterogeneous opacities with air bronchograms in bilateral lung fields and bilateral pleural effusion, left more than right

A previous chest x-ray revealed heterogeneous opacities in bilateral lung fields with bilateral pleural effusion, left more than right (Figure 1).

**Figure 2:**
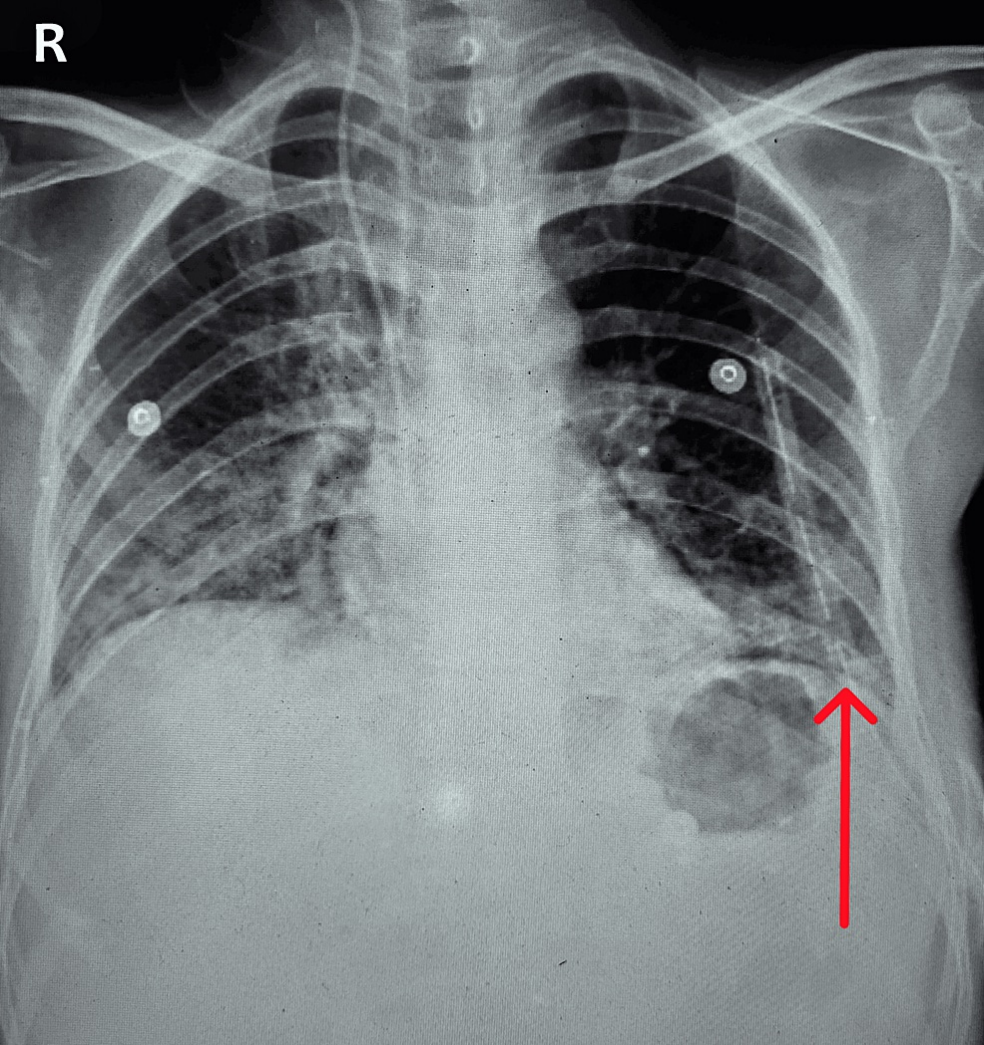
Bilateral lung field haziness on chest x-ray with left pleural effusion and left side ICD in situ

The patient had a left-sided intercostal drainage (ICD) in place and was admitted to the ICU due to respiratory distress requiring non-invasive ventilatory (NIV) support, as well as bilateral lung field haziness on chest x-rays (Figure 2).

**Figure 3:**
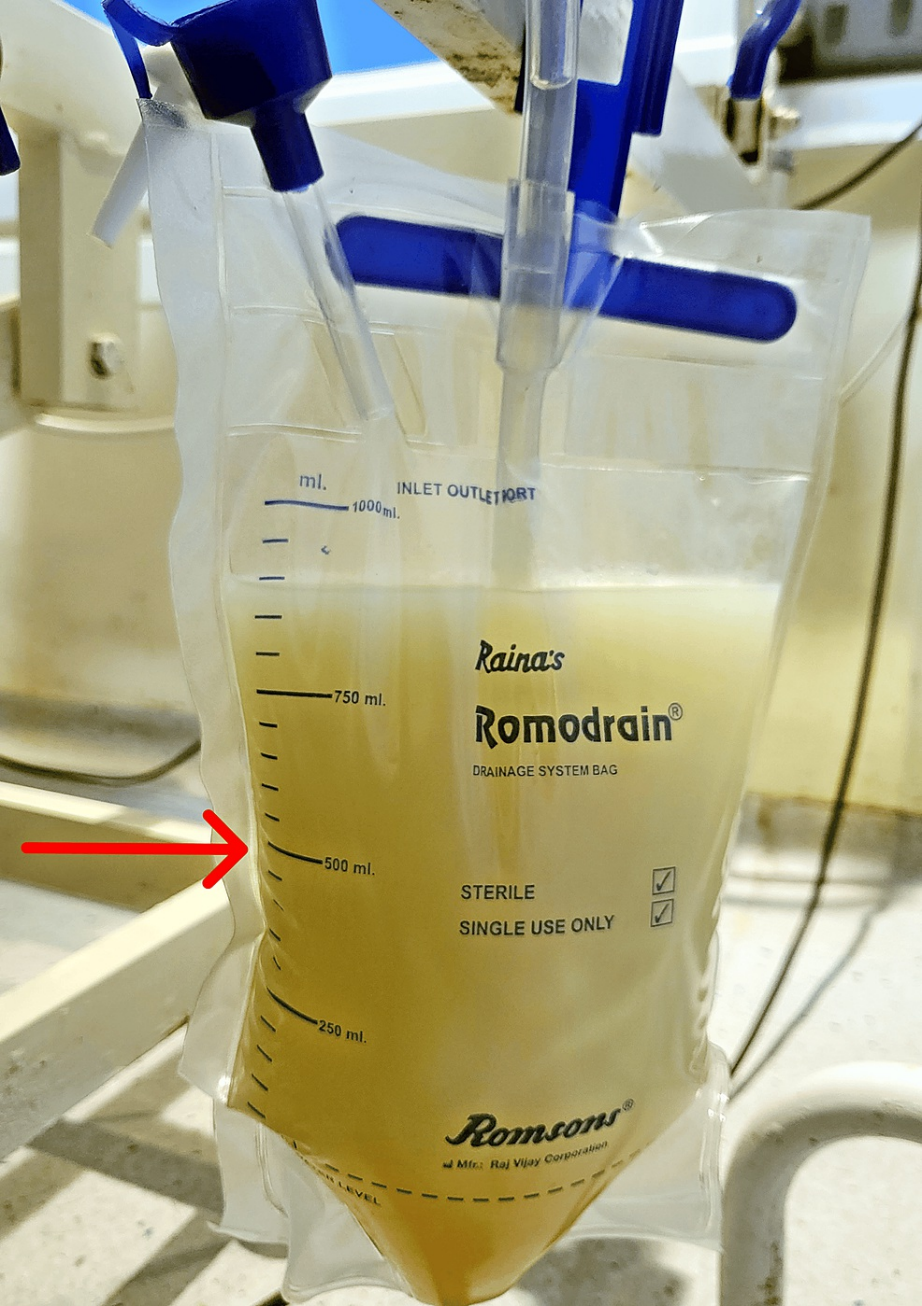
Milky white appearance of pleural fluid; chylothorax

Diagnostic thoracentesis and paracentesis were performed to verify the nature of the fluid. The pleural fluid was milky white in appearance (Figure 3).

Initial laboratory (Table 1), pleural fluid, and ascitic fluid results were as follows (Table 2). 

**Table 1:** Initial laboratory results SGOT: serum glutamic-oxaloacetic transaminase; SGPT: serum glutamic pyruvic transaminase

Lab Parameter	Result	Reference Range
White Blood Cell Count (cells/mm3)	4,700	4,000 - 11,000
Hemoglobin (g/dL)	10.4	13.8 - 17.2
Platelets (cells/mm3)	247,000	150,000 - 450,000
Serum Total Protein (g/dL)	4.6	6.0 - 8.0
Albumin (g/dL)	2.2	3.5 - 5.0
SGOT (U/L)	39	0 - 40
SGPT (U/L)	45	0 - 41
Total Bilirubin (mg/dL)	0.6	0 - 1.2
Creatinine (mg/dL)	0.6	0.6 - 1.3
Urea (mg/dL)	11	7 - 20

**Table 2:** Pleural fluid and ascitic fluid results SGOT: serum glutamic-oxaloacetic transaminase; SGPT: serum glutamic pyruvic transaminase; ZN: Ziehl Neelsen; CBNAAT: cartridge-based nucleic acid amplification test; MTB: Mycobacterium tuberculosis

Pleural Fluid Parameter	Result
Colour (Visual)	Milky white
pH	7.3
Protein Level (g/dL)	2.0
Lactate Dehydrogenase (LDH) (IU/L)	279
Triglyceride (mg/dL)	353
Cholesterol (mg/dL)	25
Adenosine Deaminase (ADA) (IU/L)	42
LDH Ratio (Fluid/Serum)	3.7
Protein Ratio (Fluid/Serum)	0.66
Cytology	Inflammatory cells seen, no malignant cells
Culture and Sensitivity	No growth
ZN Staining	MTB not detected
CBNAAT	MTB not detected
Ascites Parameter	Result
Appearance (Visual)	Milk-like and turbid
pH	7.5
Protein Level (g/dL)	2.0
Triglyceride (mg/dL)	118
Adenosine Deaminase (ADA) (IU/L)	8
Cytology	Inflammatory cells seen, no malignant cells
Culture and Sensitivity	No growth
Zn Staining	MTB not detected
CBNAAT	MTB not detected

Ultrasonography of the abdomen demonstrated free fluid in the perihepatic, perisplenic, and pelvic areas, along with hepato-splenomegaly. The color Doppler of thoracic vessels and carotid vessels showed good flow and normal caliber of all veins and arteries.

**Figure 4:**
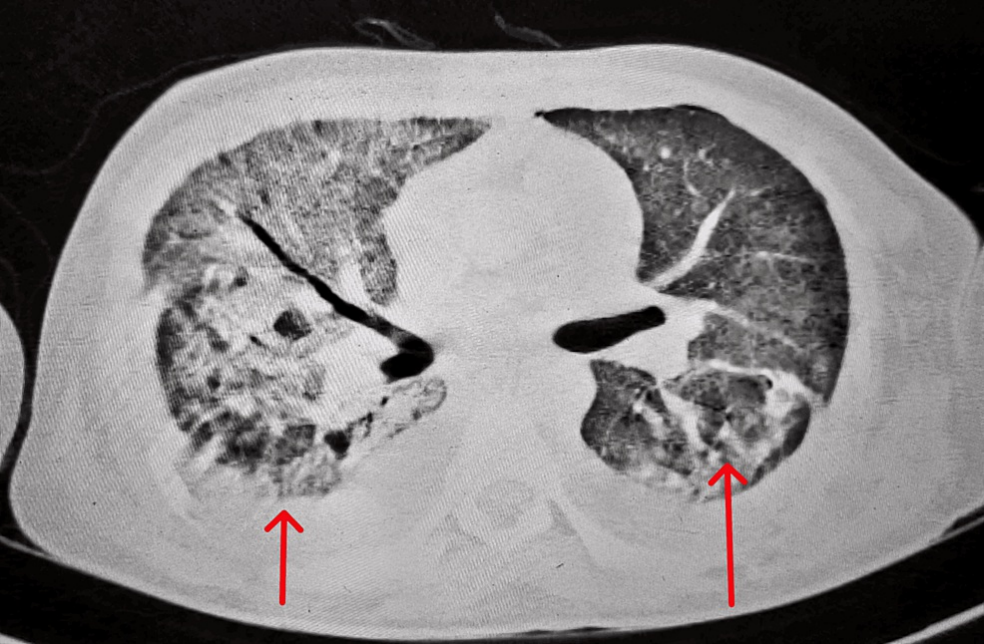
Extensive ground glass opacities in all segments of bilateral lung fields with thickening of interlobular septae at places consistent with acute respiratory distress syndrome

The computed tomography of the thorax study showed extensive ground glass opacities in all segments of bilateral lung fields, with thickening of interlobular septae at places consistent with acute respiratory distress syndrome (Figure 4).

**Figure 5:**
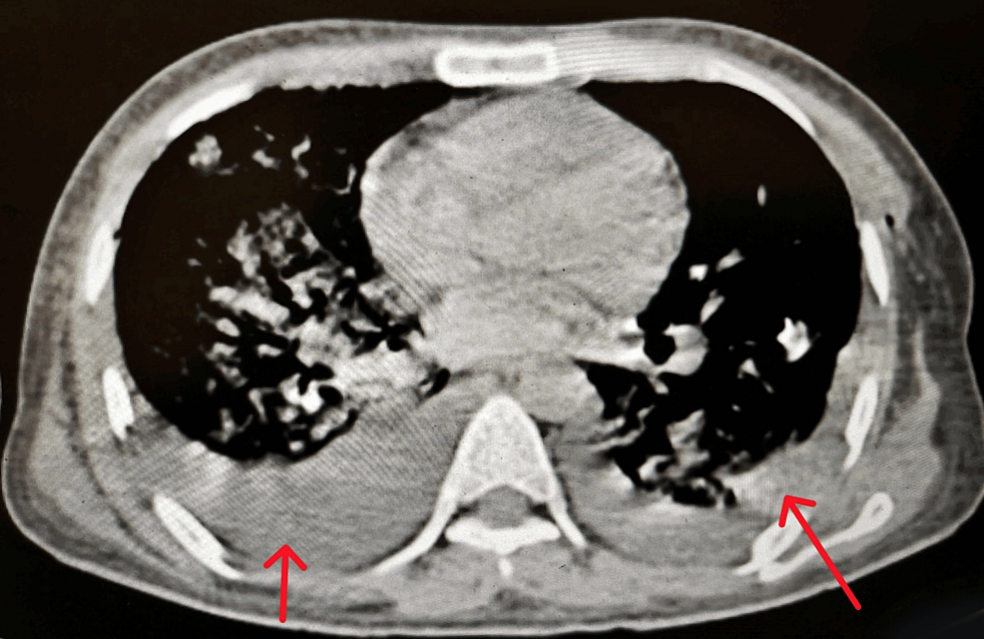
Mediastinal window of computed tomography showing bilateral moderate pleural effusion with collapse consolidation of underlying pulmonary parenchyma and bilateral basal atelectasis

Moderate pleural effusion on the left side and mild effusion on the right side with collapse consolidation of the underlying pulmonary parenchyma and bilateral basal atelectasis (Figure 5).

Computed tomography of the abdomen revealed multiple enlarged necrotic and reactive conglomerated retroperitoneal lymph nodes. Mild hepatosplenomegaly was also seen, along with moderate ascites. Hence, the gastroenterologist’s opinion was taken, and an endoscopic ultrasound (EUS)-guided biopsy was taken from the paraaortic lymph node, which came positive for Mycobacterium tuberculosis (MTB) in Gene X-pert. Also, the cytology from the lymph node showed necrotizing granuloma.

**Figure 6:**
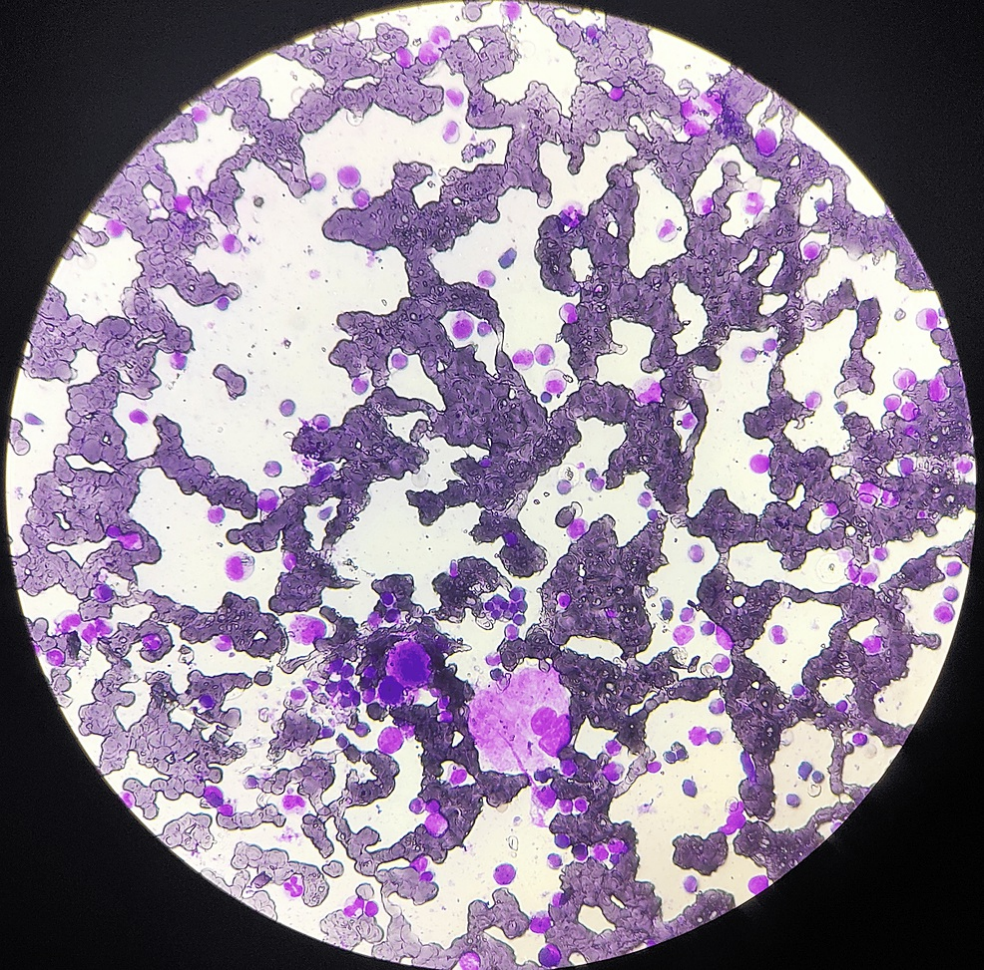
Reactive bone marrow changes with myeloid prominence, raising suspicion of hemophagocytic lymphohistiocytosis (HLH)

A bone marrow biopsy was also done, showing histiocytes with phagocytosed debris, which gave the impression of reactive bone marrow changes with myeloid prominence, raising suspicion of hemophagocytic lymphohistiocytosis (HLH) (Figure 6).

**Figure 7:**
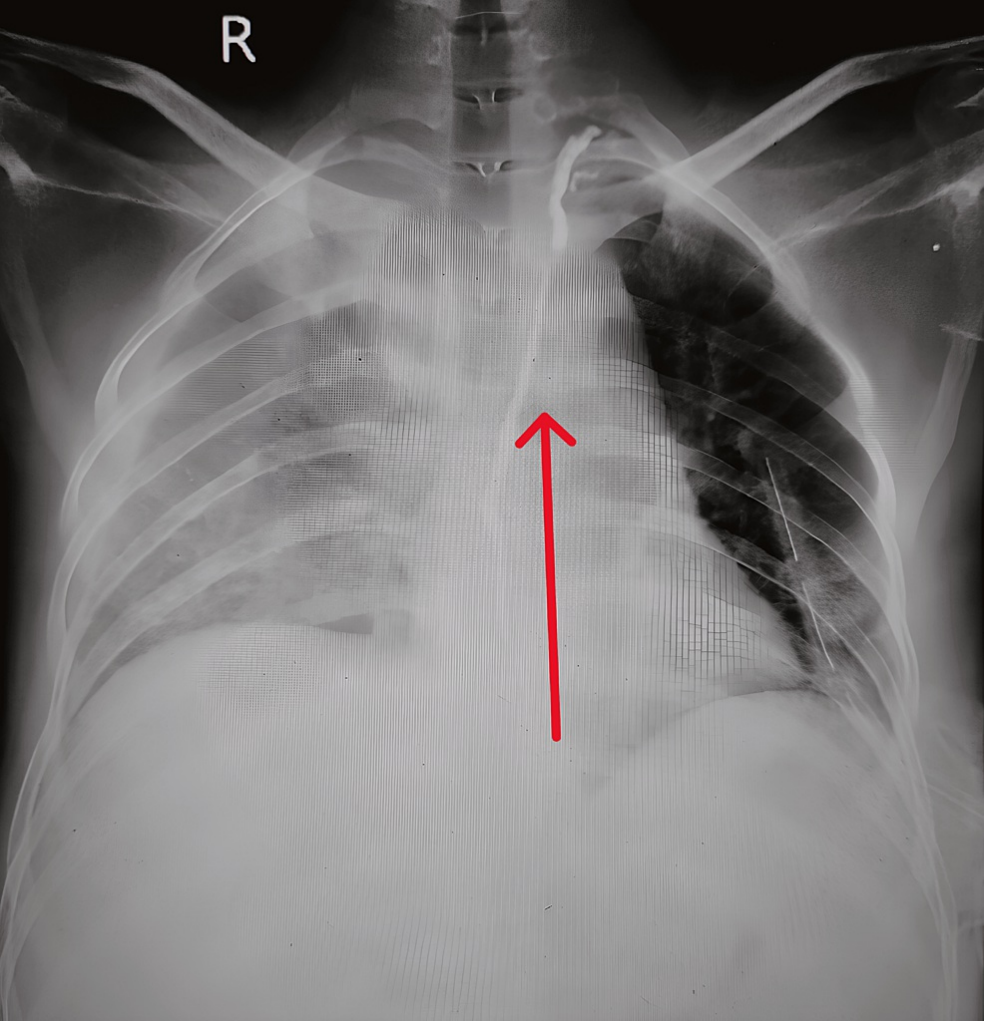
Chest x-ray anterior-posterior view post-TDE, where the embolization of the thoracic duct by cyanoacrylate glue can be seen (red arrow) TDE: thoracic duct embolization

The patient was started on anti-Koch’s therapy (isoniazid, rifampicin, pyrazinamide, and ethambutol) given the raised pleural fluid adenosine deaminase (ADA) levels and paraaortic lymph node biopsy positive for MTB. The gastroenterologist also recommended octreotide therapy of 50 micrograms three times daily for three months. Percutaneous thoracic duct embolization (TDE) was performed to address persistent drainage (Figure 7).

Given HLH on a bone marrow examination, a hepatologist’s consultation was taken, and hepatitis B virus (HBV) vaccination was advised.

**Figure 8:**
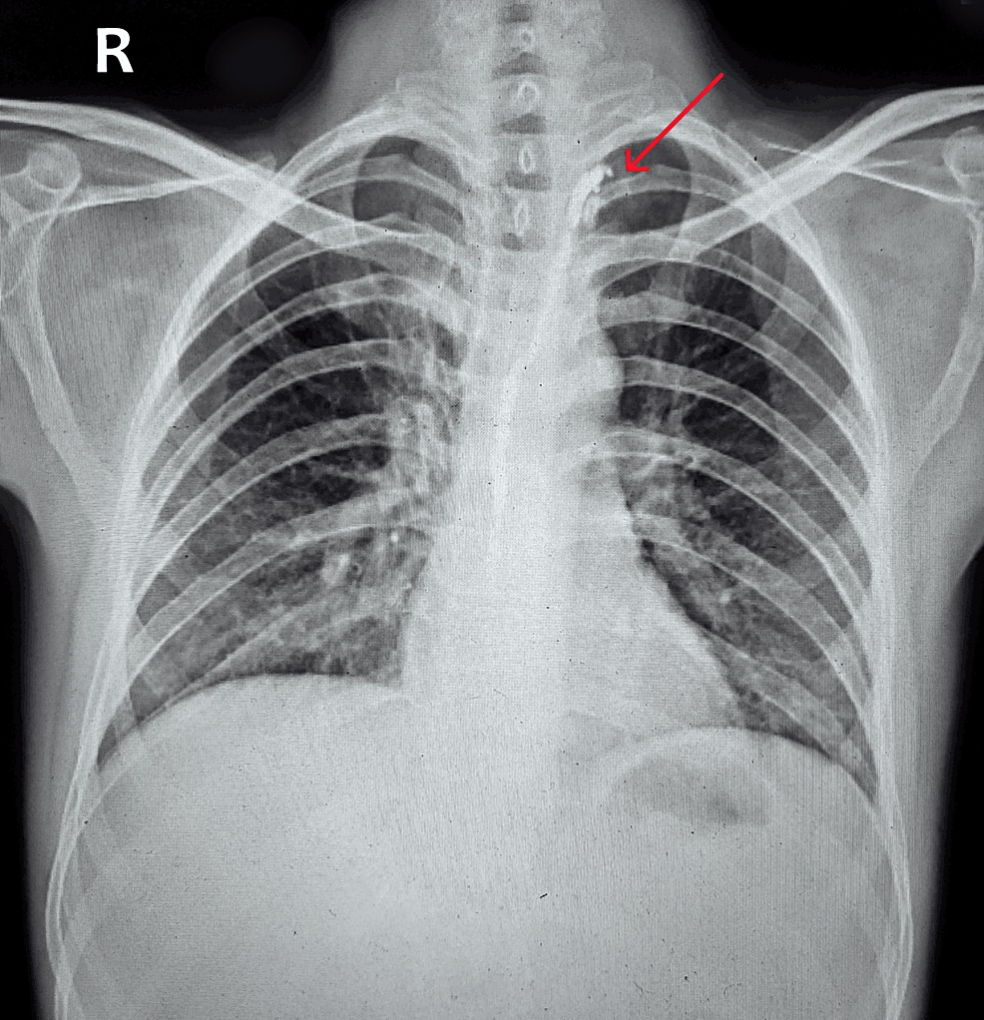
A chest x-ray anterior-posterior view post-discharge shows complete clearance of bilateral lung fields with bilaterally clear costophrenic angles indicating complete clearance of the chylothorax, with embolization of the thoracic duct (red arrow)

The patient experienced a gradual alleviation of symptoms, accompanied by a decrease in the daily drainage from the chest tube and weight gain. Consequently, the continuation of anti-tuberculosis treatment (ATT) and octreotide, along with recommended dietary adjustments, was advised. The intercostal drainage (ICD) was eventually removed upon reduced drainage and improvement observed in consecutive chest x-rays, leading to the patient's discharge in a stable condition (Figure 8).

Our patient is in continuous follow-up, as it is essential to continuously check the patient's nutritional status, recurrence, treatment reaction, and possible side effects. For HLH, the patient is being closely observed with regular follow-ups, and blood parameters are to be monitored. Depending on the patient's development, the treatment plan may need to be adjusted and regular follow-ups made.

## Discussion

The cisterna chyli is a pouch that sits in front of the second lumbar vertebra, created by the joining of lymphatic vessels from the intestines. The cisterna chyli gives rise to the thoracic duct (TD), which is roughly 36-45 cm long and 2-3 mm broad. The TD travels next to the oesophagus on the right side of the hemithorax, between the azygous vein and the aorta. It makes an arch at the seventh cervical vertebra, crosses to the left side close to the fifth or sixth vertebra, leaves the thoracic cavity at the superior thoracic aperture, and finally descends to end at the point where the left subclavian and internal jugular veins meet. Every day, the intestines transfer about 2400 milliliters of chyle to the systemic circulation. Thus, any injury, breakage, or dysfunction of the thoracic duct (TD) may result in chylothorax, which is defined as an abnormal build-up of fluid in the pleural cavity [2].

The manifestation of chyle within the pleural cavity, stemming from the impediment or breach of the thoracic duct, stands as the distinctive feature of chylothorax. The validation of this diagnosis hinges on the identification of chylomicrons and an indicative triglyceride concentration exceeding 110 mg/dL. However, it's essential to acknowledge that such benchmarks encompass a diverse spectrum of clinical conditions [2].

Staats et al. established biochemical diagnostic criteria for chylothorax in 1980. As per their research, there was a 1% probability of pleural fluid triglyceride levels above 110 mg/dL being non-chylous, but levels below 50 mg/dL had a 5% likelihood of being chylous. Therefore, the diagnosis of chylothorax relies on pleural fluid triglyceride levels exceeding 1.24 mmol/L (110 mg/dL) and cholesterol levels below 5.18 mmol/L (200 mg/dL). Conversely, elevated cholesterol levels (>5.18 mmol/L; 200 mg/dL) and diminished triglyceride levels (<0.56 mmol/L; 50 mg/dL) indicate pseudochylothorax, commonly referred to as cholesterol pleural effusion, a disorder that is frequently accompanied by the presence of cholesterol crystals [3].

**Figure 9:**
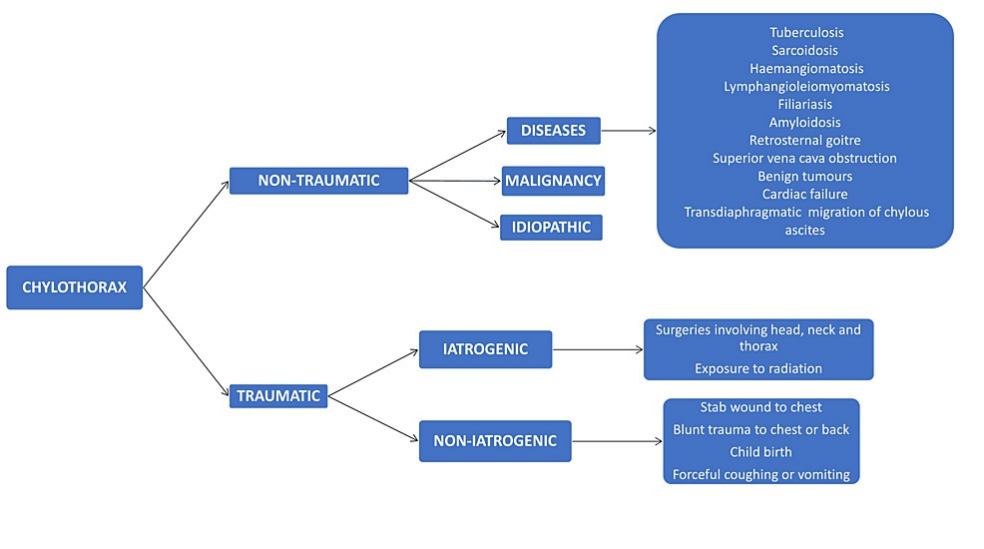
Aetiologies of chylothorax Author-created image

The etiologies of chylothorax can be broadly categorized into traumatic and non-traumatic causes. Under non-traumatic, we further have malignancy, diseases (sarcoidosis, tuberculosis, amyloidosis, superior vena cava obstruction, etc.), and idiopathy [4] (Figure 9).

TB lymphadenopathy is an infrequent etiology of chylothorax, occurring in both immunocompetent and immunocompromised individuals. Interestingly, it can manifest without mediastinal lymphadenopathy in almost half of the cases. In about 10.8% of patients, TB-induced chylothorax coexists with chylous ascites. A majority of TB-chylothorax cases respond well to anti-Koch’s treatment and adjustments in diet, with only 17.1% requiring surgical ligation of the thoracic duct [5,6].

For traumatic chylothorax, percutaneous thoracic duct embolization (TDE) has proven to be a less invasive and highly effective option compared to conservative or surgical approaches [7]. However, in our case, even though non-traumatic TDE was done, it yielded good results. In cases of high-output or medically uncontrollable chylothorax, percutaneous treatment through TDE was successful in over 70% of instances. It is recommended, especially for critically ill patients, as a preferable option before considering the more hazardous surgical ligation of the thoracic duct [8].

Hemophagocytic lymphohistiocytosis (HLH) and its cognate syndromes represent infrequent yet severe maladies intricately linked to a diverse array of infectious agents (including EBV-related conditions, sepsis, typhoid fever, tuberculosis, and leishmaniasis), in addition to genetic, neoplastic, and autoimmune disorders. In instances where HLH arises amid infection, there is a clonal surge in T-lymphocytes coupled with hyperactivation of macrophages. This syndrome poses a diagnostic challenge, resembling T-cell lymphoma, necessitating vigorous intervention with etoposide-centric chemotherapeutic protocols. Conversely, hemophagocytic syndromes intertwined with infectious etiologies such as sepsis, typhoid fever, tuberculosis, and leishmaniasis may ameliorate with targeted treatment of the underlying infection. Distinguishing these conditions is crucial due to their tendency to mimic malignant diseases [9].

## Conclusions

The coexistence of tuberculosis (TB) with manifestations like chylothorax and chylous ascites is exceptionally rare. In India, where TB prevalence is high, healthcare practitioners should consider TB in the differential diagnosis for cases involving chylothorax or chylous ascites. The management of these conditions primarily focuses on conservative measures and addressing root causes. Treatment often involves a multifaceted approach, including thoracic duct embolization (TDE) and anti-tubercular therapy. Initiating anti-tubercular medication is crucial, even in the absence of microbiological evidence, when tuberculosis is suspected. A comprehensive approach, involving collaboration among pulmonologists, gastroenterologists, intervention radiologists, nutritionists, and other healthcare professionals, is vital for successful outcomes in complex cases involving TB and chyle-related complications.
